# Factors Influencing Brucellosis Preventive Behaviors among Marginalized Iranian Women: An Application of the Health Belief Model

**DOI:** 10.1155/2022/5987484

**Published:** 2022-12-14

**Authors:** Majid Barati, Rezvan Shayghan Zahed, Marjan Bakhtiari, Yadollah Fathi, Maryam Afshari, Zahra Taheri-Kharameh

**Affiliations:** ^1^Social Determinants of Health Research Center, Hamadan University of Medical Sciences, Hamadan, Iran; ^2^Department of Public Health, Asadabad School of Medical Sciences, Asadabad, Iran; ^3^Students Research Committee, Hamadan University of Medical Sciences, Hamadan, Iran; ^4^Department of Public Health, School of Health, Hamadan University of Medical Sciences, Hamadan, Iran; ^5^Research Center for Health Sciences, Hamadan University of Medical Sciences, Hamadan, Iran; ^6^School of Paramedical Sciences, Qom University of Medical Sciences, Pardisan, Iran

## Abstract

**Background:**

Brucellosis is still a serious public health threat in developing countries, especially in Iran. Brucellosis is an endemic disease in Iran and risk factors increasing its broadcast are prevalent. This study investigated factors influencing brucellosis preventive behaviors amongst marginalized women in Hamadan city, Iran, using the health belief model (HBM).

**Methods:**

In this cross-sectional study, 289 women living in marginal areas were considered from April until May 2018. Via cluster random sampling methods, comprehensive health services where women get health care were selected. Each woman in the selected comprehensive health services was then enrolled by the simple random sampling method. Data were gathered from a face-to-face interview via a questionnaire.

**Results:**

Most women had a history of using nonpasteurized dairy products (86.2%). Most women (64.7%) boiled the milk for 3–5 minutes. 61.2% of women put the cheese in a salt-water solution. The results showed that one unit rise in the scores of knowledge, self-efficacy, and cues to action resulted in an increase in the mean score of the protective behavior by 0.189, 209, and 0.150, respectively.

**Conclusion:**

The HBM was a helpful model in predicting the influential elements in brucellosis preventive behaviors. Also, recognized effective factors should be taken into account when designing interventions.

## 1. Introduction

Brucellosis is one of the essential zoonotic diseases and it is a major challenging issue for human health worldwide due to the physical suffering and reduced work ability of those infected, along with the reduced livestock productivity [1]. It may reason for an extensive spectrum of medical symptoms in individuals, from mild disease to severe illnesses [2]. According to the World Health Organization report, specifically in developing countries, brucellosis is one of the seven neglected zoonotic diseases [3]. Contrary to the excessive improvement in the care and eradication of brucellosis in the majority of countries, there are yet areas with great prevalence rates where the infection continues among domestic animals and, as a result, regular transmissions happen in the populations at risk [4].

Iran is an endemic area for brucellosis with a variety of 0.5% to 10.9% for the prevalence rate in different regions [4]. In this country, the province with the highest prevalence of brucellosis was reported to be Hamadan province [5]. So, the transmission of brucellosis occurs in contact with domestic animals or their products and through occupational exposure [6]. Also, brucellosis diseases have been displayed to increase with individual age and to be increased in women [7]. So, López-Merino's study showed that the disease is more prevalent in females than in males and found more at 25–64 years of age than in other age groups [8]. Women at the city's margins are more at risk [9]. Marginal settlement is one of the chief problems in cities, especially in big cities, which may harmfully affect an individual's health. Furthermore, according to the literature, women living in these areas have more problems, such as unsuitable living environments, deprivation from health facilities, and poverty [10].

In Iran, behavior change models and theories have usually been used to report diverse health subjects [4, 11–13]. Nevertheless, limited studies have applied the models to predict preventive behaviors for infectious diseases such as brucellosis [14]. One of the current theories in health education is the health belief model (HBM), which is extensively recognized as a context for the expectation of preventive behaviors [15]. HBM emphasizes how the perceptions and motivations of an individual cause behavior in them. Generally, the model emphasizes changes in beliefs, and differences in assumptions will cause di in behavior and have construct perceived susceptibility, perceived severity, benefits perceived, barriers perceived, cues to action, and self-efficacy [15, 16].

To the best of our knowledge, little evidence is known about the related factors for brucellosis prevention behavior in marginalized women, and there have been no studies that included Iranian marginalized women. This study was conducted to recognize the influential determinants of Brucellosis preventive behaviors based on the health belief model in marginalized Iranian women in Hamadan city, Iran.

## 2. Materials and Methods

### 2.1. Settings

In this cross-sectional study, 289 women living in marginal areas of Hamadan city in western Iran were studied from April to May 2018. Hamadan city is one of the central provinces in the marginalization subject in Iran. Hamadan city has eleven marginal areas [17].

### 2.2. Study Sample

By random cluster sampling method, marginalized women were selected from those covered by 11 comprehensive health services in marginal areas of Hamadan city. Then, the quota for each comprehensive health service was estimated from the number of samples according to the population covered. Finally, each woman within the selected comprehensive health services was then enrolled in the study of simple random sampling.

Taking into consideration the sample size in the Khanian and Hashemian's study [18], the maximum standard deviation of protective behaviors on brucellosis was equal to 1.5, with an acceptable error of 0.5 and a confidence interval of 95%, and design effect for cluster random sampling was taken as 1.5, which showed that the sample size was 289 women.

### 2.3. Data Collection

Data were gathered from a face-to-face interview on an author-developed questionnaire that involved two parts including (a) demographic data of age, marital status, educational status, women's job and husband's job, history of residence in the village, travel to rural areas, and history of brucellosis in family members and use of nonpasteurized dairy products; (b) the second part of the questionnaire consisted of questions about knowledge and HBM constructs. Interviews were conducted in a private room of the comprehensive health services, and interview time was scheduled based on the participants' convenience.

Questions of knowledge (21 items) had two options, such as yes and no. The scoring method for questions was such that questions, which related to knowledge of the correct answer to them, were given one score and the wrong answer a score of 0, so that all of these points could vary from 0 to 21 (e.g., is it possible to prevent brucellosis in humans?)

The perceived susceptibility (4 items), perceived severity (7 items), benefits perceived (4 items), barriers perceived (6 items), and cues to action (6 items) constructs were determined by a Likert scale as follows: fully agree (5 points), agree (4 points), no idea (3 points), disagree (2 points), and fully disagree (1 point). Therefore, the choice of possible points for the perceived susceptibility construct was 4 to 20 (e.g., it is possible that I will get brucellosis if I use local cheese), the construct of perceived severity was 7 to 35 (e.g., if I get brucellosis, I have to endure a lot of pain), benefits perceived were 4 to 20 (e.g., pasteurized dairy products increase my health), barriers perceived were 6 to 30 (e.g., treatment for brucellosis is lengthy and costly), and cues to action were 6 to 30 (e.g., my family encourages me to eat pasteurized dairy products). The self-efficacy (6 items) constructs were measured by the Likert scale as from very little (1 point), a little (2 points), to some extent (3 points), much (4 points), and very much (5 points). Thus, the range of possible points and sample questions for these constructs of self-efficacy was 6 to 30 (e.g., I can use pasteurized milk).

The constructs of brucellosis prevention behavior (twelve items) were measured on the Likert scale: never (1 point), sometimes (2 points), and always (3 points). The range of potential points for this variable was 12 to 36 (e.g., I use pasteurized milk).

### 2.4. Pilot Testing of Questionnaire

The draft of the questionnaire was developed and determined by items as determined in the study and according to the experiences of the investigators. Then, content and face validity and Cronbach's alpha coefficient were determined by analyzing the pilot data. To assess the content validity, the questionnaire was reviewed by a panel of experts, nine health education and promotion and occupational health professionals. The content validity ratio (CVR) was calculated through experts' opinions; items with a score of 0.78 or more remained in the questionnaire. The content validity index (CVI) was calculated by experts on a four-point scale. A score of 0.79 was considered as the least suitable CVI. The assessed face validity questionnaire was measured by interviewing 10 women. This pilot study proposed minor changes to the questionnaire before finalization.

Its reliability was examined by conducting a pilot study on 30 marginalized women, and Cronbach's alpha coefficient was documented as 0.73, 0.77, 0.81, 0.82, 0.71, 0.79, and 0.75 for perceived susceptibility, perceived severity, benefits perceived, barriers perceived, cues to action, self-efficacy, and prevention behavior constructs, respectively.

### 2.5. Ethical Approval

An informed consent was obtained from the women after describing the goal of the study. The Ethical Committee of Hamadan University of Medical Sciences approved this study.

### 2.6. Data Analysis

Data analysis was performed using SPSS 24. Inferential statistics including Pearson correlation coefficient, ANOVA, independent *t* tests, and linear regression analysis were employed to determine the relationship between variables. The significance level of the tests was set at 5%.

## 3. Results

However, 289 women participated in the study and the response rate was equal to 100%. As shown in Table 1, the highest percent of the age group was related to 21–30 years old (41.5%), and their highest education level was a diploma (30.8%). In addition, most women were married (89.3%). 92% of the women were housewives, and their husband's job was free (46.2%). More than half of the women had a history of residence in the village (73.7%). About 82% of the participants traveled to rural areas. In addition, 89.3% did not report a history of brucellosis in their relatives. Furthermore, 86.2% of the women had a history of the use of nonpasteurized dairy products.

The results showed a positively correlated *r* = 0.372, *P* < 0.01, between perceived susceptibility and knowledge of getting brucellosis. Moreover, the perceived severity of getting brucellosis had a positive correlation with knowledge (*r* = 0.344, *P* < 0.01 1) and perceived susceptibility (*r* = 0.448, *P* < 0.01). Furthermore, the benefits perceived in adopting prevention behavior had a positive correlation with knowledge (*r* = 0.134, *P* < 0.05) and perceived severity (*r* = 0.225, *P* < 0.01). There was a positive correlation between self-efficacy and benefits perceived (*r* = 0.316, *P* < 0.01). Also, the cues to action had a positive correlation with perceived severity (*r* = 0.230, *P* < 0.01). Finally, there was a positive correlation between the prevention behavior of brucellosis and self-efficacy (*r* = 0.319, *P* < 0.01) and cues to action (*r* = 0.268, *P* < 0.01) (Table 2).

As shown in Table 3, 46% reported regular use of pasteurized milk, 69.9% always use taped milk, 31.1% always use fresh cheese, 85.8% always use uncooked or semicooked meat, and 49.1% always use boiled or steaming liver. Most women (64.7%) boiled the milk for 3–5 minutes.

Moreover, 59.9% of women use pasteurized peanuts, cream, and ice cream. Among all respondents, 61.2% of the women kept cheese in salt-water solution, 92.7%, 94.5%, 43.6%, and 42.9% clearing the vegetable from mud and soil, washing the vegetables with clean water, using dishwashing liquid to clean vegetables, and using the antiseptic solutions to wash vegetables, respectively.

As presented in Table 4, perceived susceptibility had a significant relationship with age and marital status (*P* < 0.05). There was also a significant relationship between perceived severity and education status (*P* < 0.05). In addition, there was a significant relationship between the benefits perceived and women's jobs (*P* < 0.05). Self-efficacy had a significant association with education status (*P* < 0.05). Moreover, prevention behaviors had a significant association with age, education status, and husband's job (*P* < 0.05). However, the other constructs of the HBM had no statistically significant association with the demographic and background variables (*P* > 0.05).

As shown in Table 5, one unit increase in the knowledge score was associated with the mean score of prevention behavior increased by 0.189. Also, with one-unit increase in self-efficacy score, the mean score of the behavior increased by 0.209. In addition, one-unit increase in the cues to action score was associated with the mean score prevention behavior increased by 0.150.

## 4. Discussion

The total burden of brucellosis remains widespread [19]. Although eradicated in the majority of developed countries after enormous effort, the infection is still a key neglected disease in Iran as a developing country. Addressing the issue of analyzing the preventive behaviors of brucellosis is one of the main issues in the control and management of diseases and specifically brucellosis disease among residents of urban areas. Identifying related factors in this group is one of the most important research priorities in this section.

More than half of the women had a history of residence in the village (73.7%). Most women had a history of using nonpasteurized dairy products (86.2%). Most women (64.7%) boiled the milk for 3–5 minutes. 61.2% of the women put the cheese in a salt-water solution. The results showed that with one unit rise in the scores of knowledge, self-efficacy and cues to action resulted in an increase in the mean score of the protective behavior by 0.189, 209, and 0.150, respectively.

The results of this study showed that the majority of women had a history of using local dairy products. The obtained results were similar to the findings of a previous study [20, 21]. Probably, these products tend to be delicious compared to other dairy products and are individually tested.

Our findings exposed that women implement protective behaviors properly. This result is not constant with the results of Chagunda et al. [22] and Igawe et al.'s study [3]. Usually, the support of public health prevention works on the hypothesis that better knowledge and other factors, including perceived susceptibility, perceived severity, benefits perceived, barriers perceived, cues to action, and self-efficacy cues for protective behaviors in women. The strategy is to increase knowledge and proper belief that women will be involved in behaviors.

In this study, the brucellosis preventive behaviors were significantly correlated with age, education status, and husband's job. This result is constant with the results of previous studies [23–25]. Generally, older people show better behaviors because of experience. In addition, those who have a higher education status and whose husbands have better jobs have better health behaviors due to proper economic conditions.

During the construct investigation, it was found that knowledge, self-efficacy, and cues to action explained about 15% of the total variance. This finding approves the HBM model, which has a significant role in performing healthy behavior, especially in brucellosis protective behavior. Those results are constant with the results suggested in the previous studies [4, 26, 27]. Also, self‐efficacy is a key precondition for self‐management in promoting preventive behaviors [14, 28].

The results of this study should be understood in light of two limitations. Firstly, self-reported data on behavior prevention and other factors may introduce potential bias. Secondly, we used a cross-sectional study design.

## 5. Conclusion

The health belief model was found as a helpful model in predicting the effective factors in brucellosis preventive behaviors. Prevention and intervention programs should recognize the factors of brucellosis prevention in marginalized women; in addition, the recognized factors must be taken into account when designing interventions and implementing them. Also, other studies with this model can be conducted in other places and with other target groups to obtain more specific results.

## Figures and Tables

**Table 1:** Demographic and background characteristics of the participants (*n* = 289).

Characteristics	*N*	Percent
*Age* (*year*)		
≤20	18	6.2
21–30	120	41.5
31–40	108	37.4
41–50	26	9.0
≥51	17	5.9

*Education status*		
Illiteracy	41	4.9
Elementary school	65	22.5
Middle school	40	13.8
Diploma	89	30.8
≥ college	81	28.0

*Marital status*		
Married	258	89.3
Single	18	6.2
Divorced or widowed	13	4.5

*Women*'*s job*		
Housewife	266	92.0
Working	23	8.0

*Husband*'*s job*		
Farmer or laborer	80	31.0
Working	54	20.9
Free	119	46.2
No husband	31	10.7

*History of residence in the village*		
Yes	213	73.7
No	76	26.3

*Travel to rural areas*		
Yes	237	82.0
No	52	18.0

*History of brucellosis in family members and friends*		
Yes	31	10.7
No	258	89.3

*History of use of nonpasteurized dairy products*		
Yes	249	86.2
No	40	13.8

**Table 2:** The correlation coefficient matrix of health belief model (*n* = 289).

Construct	Knowledge	Perceived susceptibility	Perceived severity	Benefits perceived	Barriers perceived	Self-efficacy	Cues to action	Prevention behaviors
Knowledge	1							
Perceived susceptibility	0.372^*∗∗*^	1						
Perceived severity	0.344^*∗∗*^	0.448^*∗∗*^	1					
Benefits perceived	0.134^*∗*^	0.081	0.225^*∗∗*^	1				
Barriers perceived	0.048	0.097	0.201	−0.005	1			
Self-efficacy	0.196	0.096	0.206	0.316^*∗∗*^	−0.164	1		
Cues to action	0.088	0.064	0.230^*∗∗*^	0.180	−0.088	0.088	1	
Prevention behaviors	0.243	0.076	0.185	0.168	0.001	0.319^*∗∗*^	0.268^*∗∗*^	1

^*∗*^ *P* < 0/05, ^*∗∗*^*P* < 0/ 01.

**Table 3:** Women's answers to questions about brucellosis prevention behaviors (*n* = 289).

Questions	Never		Sometimes		Always	
	*N*	%	*N*	%	*N*	%
Use of pasteurized milk	26	9.0	130	45.0	133	46.0
Use of the taped milk	21	7.3	66	22.8	202	69.9
Use of fresh cheese	113	39.1	86	29.8	90	31.1
Use of uncooked or semicooked meat	11	3.8	30	10.4	284	85.8
Boiling or steaming liver	56	19.4	91	31.5	142	49.1
Boiling the milk for 3–5 minutes	60	20.8	42	14.5	187	64.7
Use of pasteurized peanut, cream, and ice cream	22	7.6	94	32.5	173	59.9
Maintenance of cheese in salt-water solution	50	17.3	62	21.5	177	61.2
Clearing the vegetable from mud and soil	3	1.0	18	6.2	268	92.7
Washing the vegetables with clean water	3	1.0	13	4.5	273	94.5
Using dishwashing liquid to clean vegetables	77	26.6	86	29.8	126	43.6
Using the antiseptic solution to wash vegetables	79	27.3	86	29.8	124	42.9

Note: *N* = number; % = percent.

**Table 4:** The relationship of Health Belief Model with demographic and background characteristics (*n* = 289).

Variables	Mean	SD	95% CI		*P* value
			Lower	Upper	
*Perceived susceptibility*					
*Age* (year)					
≤20	9.83	2.91	9.21	10.2	0.015
21–30	10.10	2.19	9.46	10.30	
31–40	10.47	2.51	10.1	10.88	
41–50	10.69	2.18	10.12	11.01	
≥51	12.36	2.51	12.03	12.63	
*Marital status*					
Married	10.31	2.41	10.01	10.78	0.039
Single	10.89	2.34	10.23	11.21	
Divorced or widowed	12.30	1.49	12.02	12.46	
*Perceived severity*					
*Education status*					
Illiteracy	27.86	2.07	27.23	28.12	0.025
Elementary school	24.65	4.34	24.12	25.10	
Middle school	25.42	4.41	25.05	25.67	
Diploma	26.02	4.40	25.38	26.56	
≥ college	25.12	3.87	24.89	25.45	
*Benefits perceived*					
*Women*'s job					
Housewife	15.43	3.18	15.14	15.88	0.016
Working	17.09	2.69	16.85	17.32	
*Self-efficacy*					
*Education status*					
Illiteracy	15.14	4.13	14.78	15.64	0.003
Elementary school	17.93	3.37	17.13	18.41	
Middle school	18.33	3.53	18.14	18.65	
Diploma	18.88	3.74	18.25	19.10	
≥ college	16.44	3.32	16.15	16.77	
*Prevention behaviors*					
*Age* (year)					
≤20	23.28	3.26	23.09	23.74	0.026
21–30	21.71	3.39	21.14	21.98	
31–40	22.67	3.52	22.16	22.79	
41–50	23.69	3.63	23.17	23.88	
≥51	24.18	4.07	23.98	24.56	
*Education status*					
Illiteracy	22.79	3.72	22.19	23.14	0.014
Elementary school	23.30	3.29	23.00	23.56	
Middle school	21.85	3.74	21.42	22.11	
Diploma	20.92	3.78	20.78	21.32	
≥College	21.91	3.44	21.74	22.30	
*Husband*'s job					
Farmer or laborer	23.14	3.23	22.89	23.43	0.010
Working	21.42	3.37	21.01	21.66	
Free	22.48	3.42	22.13	22.79	
No husband	21.90	3.77	21.46	22.37	

Note: SD = standard deviation.

**Table 5:** Predicting brucellosis prevention behavior among women: linear regression analyses (*n* = 289) (adjusted *R*^2^ = 0.144).

Construct	*β*	B	SE	95% CI		*P* value
				Lower	Upper	
Knowledge	0.189	0.196	0.058	0.082	0.309	0.001
Self-efficacy	0.209	0.204	0.062	0.082	0.326	0.001
Cues to action	0.150	0.127	0.053	0.023	0.232	0.017
Constant	—	13.573	1.283	0.004	11.047	16.099

Note: *β* = beta; B = unstandardized regression coefficient; SE = standard error.

## Data Availability

The data used in this study are available on request from the corresponding author.
